# Superior efficacy of St John's wort extract WS^® ^5570 compared to placebo in patients with major depression: a randomized, double-blind, placebo-controlled, multi-center trial [ISRCTN77277298]

**DOI:** 10.1186/1741-7015-4-14

**Published:** 2006-06-23

**Authors:** Siegfried Kasper, Ion-George Anghelescu, Armin Szegedi, Angelika Dienel, Meinhard Kieser

**Affiliations:** 1 Department of General Psychiatry, Vienna Medical University, Währinger Gürtel 18–20, A-1090 Wien, Austria; 2 Klinik und Hochschulambulanz für Psychiatrie und Psychotherapie, Charité – Universitätsmedizin Berlin, Eschenallee 3, D-14050 Berlin, Germany; 3 Clinical Research Department, Dr. Willmar Schwabe Pharmaceuticals, Willmar-Schwabe-Straße 4, D-76227 Karlsruhe, Germany

## Abstract

**Background:**

The aim of the current study was to assess the antidepressant efficacy and safety of *Hypericum perforatum *(St. John's wort) extract WS^® ^5570 at doses of 600 mg/day in a single dose and 1200 mg/day in two doses.

**Methods:**

The participants in this double-blind, randomized, placebo-controlled, multi-center clinical trial were male and female adult out-patients with an episode of mild or moderate major depressive episode (single or recurrent episode, DSM-IV criteria). As specified by the relevant guideline, the study was preceded by a medication-free run-in phase. For the 6-week treatment, 332 patients were randomized: 123 to WS^® ^5570 600 mg/day, 127 to WS^® ^5570 1200 mg/day, and 82 to placebo. The primary outcome measure was the change in total score on the Hamilton Rating Scale for Depression (HAM-D, 17-item version) between baseline and endpoint. Additional measures included the number of responders, the number of patients in remission, and several other standard rating scales. Efficacy and safety were assessed after 2 and 6 weeks. The design included an interim analysis performed after randomization with the option of early termination.

**Results:**

After 6 weeks of treatment, mean ± standard deviation decreases in HAM-D total scores of 11.6 ± 6.4, 10.8 ± 7.3, and 6.0 ± 8.1 points were observed for the WS^® ^5570 600 mg/day, 1200 mg/day and placebo groups, respectively (endpoint analysis). Secondary measures of treatment efficacy also showed that both WS^® ^5570 groups were statistically superior to placebo. Significantly more patients in the WS^® ^5570 treatment groups than in the placebo group showed treatment response and remission. WS^® ^5570 was consistently more effective than placebo in patients with either less severe or more severe baseline impairment. The number of patients who experienced remission was higher in the WS^® ^5570 1200 mg/day group than the WS^® ^5570 600 mg/day group. The incidence of adverse events was low in all groups. The adverse event profile was consistent with the known profile for *Hypericum *extract preparations.

**Conclusion:**

*Hypericum perforatum *extract WS^® ^5570 at doses of 600 mg/day (once daily) and 1200 mg/day (600 mg twice daily) were found to be safe and more effective than placebo, with comparable efficacy of the WS^® ^5570 groups for the treatment of mild to moderate major depression.

## Background

St. John's wort (*Hypericum perforatum *extract) is an attractive treatment option for patients with mild to moderately severe depression because it has been shown in clinical trials to be effective [1-6] and well tolerated with a more favorable side-effect profile than many synthetic antidepressants [2,6-10]. The use of preparations of St. John's wort extracts for patients with major depression, however, remains somewhat controversial, since some studies in major depression showed no significant effect [e.g. [11,13]]. Recent meta-analyses of randomized double-blind trials that tested the efficacy of St. John's wort extracts against either placebo or standard antidepressants [14-16] have shown the current evidence as to whether the effects are marked or minor in patients with major depression to be inconsistent and confusing.

Experimental investigations have provided evidence that serotonin receptor expression is markedly reduced during treatment with *Hypericum *extract, ultimately leading to enhanced synaptic availability of serotonin and norepinephrin [17,18].

St. John's wort preparations are extracted from the plant's flowers and leaves, harvested just before or during the flowering period. The ingredients include hypericin along with flavonoids, xanthone derivates, plant acids (chlorogenic acid, caffeic acid), tannins (catechin) and the phloroglucin hyperforin. The inhibition of neuronal uptake of serotonin and other biogenic amines and amino acid neurotransmitters is probably mainly attributable to hyperforin.

The therapeutic profile of St. John's wort extract in the treatment of mild to moderate depression is rather general, influencing all signs and symptoms of the disease, similar to the profile of serotonin uptake inhibitors [14]. Whether there is a relationship between the initial severity of the patient's depression and the efficacy of St. John's wort extract has important clinical implications. Laakmann et al. [2] investigated St. John's wort extract in mildly to moderately depressed patients with a baseline total score on the Hamilton Rating Scale for Depression (HAM-D, 17-item version) of 17 or higher. The results suggested that the more severely depressed subgroup (those with an initial total score on the HAM-D scale of 22 or higher) experienced greater antidepressant benefit from treatment with St. John's wort extract than the less severely depressed subgroup.

The safety and efficacy of the St. John's wort extract WS^® ^5570 have been successfully investigated in recent studies. Lecrubier et al. [4] demonstrated the efficacy of WS^® ^5570 at a dose of 300 mg three times daily for 6 weeks, showing it to be safe and more effective than placebo in patients with mild to moderate major depression. In addition, WS^® ^5570 300 mg three times daily was demonstrated to be at least equally effective and better tolerated than paroxetine in patients with moderate to severe depression [19].

The optimal dosage and dose regimen of St. John's wort extract have not been definitively established. A total daily dose of 2–4 g of St. John's wort has been recommended by the Commission E Monographs [20]. Most of the available clinical data have been carried out for 300 mg preparations of St. John's wort extract taken three times daily. In a postmarketing survey with 2166 patients, Rychlik et al. [21] demonstrated an improvement in depressive symptoms with 600 mg St. John's wort extract taken once or twice daily similar to that observed with 300 mg three times daily. The current study was designed to assess the antidepressant efficacy and safety of St. John's wort extract WS^® ^5570 at doses of 600 mg/day (given only once daily) and 1200 mg/day (given as 600 mg twice daily) over 6 weeks of treatment in patients suffering from a major depressive episode.

## Methods

### Protocol, design and objectives

This was a 6-week randomized, double-blind, placebo-controlled, multi-center, clinical phase III trial comparing the efficacy of St. John's wort extract WS^® ^5570 600 mg/day (in one dose) and 1200 mg/day (in two 600 mg doses) with placebo in patients suffering from a mild or moderate major depressive episode according to DSM-IV [21]. The investigation was conducted between April 2003 and August 2004 at 16 centers (11 psychiatrists, 5 general practitioners) in Germany. It followed the Good Clinical Practice guidelines of the European Union, the Declaration of Helsinki, and German regulatory and legal requirements. An independent ethics committee (EC of Landesärztekammer Baden-Württemberg, Germany) approved the trial protocol prior to study initiation (code 227-02). All patients provided written informed consent. In accordance with EMEA guidance on the performance of clinical investigations of medicinal products in the treatment of depression, the 6-week treatment period was not preceded by a placebo wash-out period and established rating scales were used to assess antidepressant efficacy. The raters were blind to treatment assignment. Responders were offered continued treatment in a 16-week double-blind maintenance phase. The primary objective was to establish the efficacy of WS^® ^5570 600 mg/day (in one dose) and 1200 mg/day (in two doses) during the 6 weeks of acute treatment. Secondary objectives were to assess the safety and tolerability of the treatments investigated. The primary outcome measure was the intra-individual change in the total score of the Hamilton Rating Scale for Depression (HAM-D, 17-item version) between baseline and the end of the 6-week acute phase. Visits took place at baseline and on days 14 and 42. The study employed an adaptive interim analysis to enable early termination if the presence or absence of treatment differences were clear, or continuation of the study to a second stage with a revised sample size.

### Participants

General practitioners and psychiatrists conducted the study. The episode was required to be of at least two weeks' but not more than one year's duration. We recruited male and female patients, 18 to 65 years of age, with a diagnosis of a mild or moderate, single or recurrent, major depressive episode as defined by the DSM-IV [22] (diagnostic codes for general practitioners: single episode 296.21, recurrent episode 296.31; for psychiatrists: single episode 296.21 or 296.22, recurrent episode 296.31 and 296.32). Participants were required to have HAM-D total score ≥18 and HAM-D item "depressive mood" ≥2 at baseline.

### Investigational treatments

Film-coated tablets of WS^® ^5570 containing 600 mg of dry extract from *Hypericum perforatum *(drug-to-extract ratio 3–7:1, extraction solvent methanol 80% v/v) were manufactured by Dr. Willmar Schwabe Pharmaceuticals, Karlsruhe, Germany. Tablets containing placebo were indistinguishable from those containing WS^® ^5570 in all aspects of their appearance. During the 6 weeks of randomized treatment, all patients took two film-coated tablets per day, one in the morning and one in the evening. Patients randomized to 600 mg St. John's wort extract took one WS^® ^5570 tablet in the morning and one placebo tablet in the evening. Doses were administered with liquid, shortly before meals. The randomization ratio was 3:3:2 for WS^® ^5570 600 mg/day, WS^® ^5570 1200 mg/day and placebo, respectively. This allocation ratio was chosen to avoid unnecessary exposure of depressed patients to placebo, to obtain more information on the active treatment group, and simultaneously to minimize the power reduction caused by unequal allocation. The Biometry Department at Dr. Willmar Schwabe Pharmaceuticals generated the randomization sequence in balanced groups of eight patients using a validated random number generator. Dr. Willmar Schwabe Pharmaceuticals packaged the test drug in numbered containers according to the randomization list and shipped them to the centers in balanced blocks of consecutive drug numbers. Upon enrollment, participants received a patient number; patients who were randomized received a drug number, sequentially allocated in the order of inclusion in the randomized treatment period within the trial centers. Each patient received only the test drug labeled with the assigned drug number. The randomization list was kept sealed in a secure location at Dr. Willmar Schwabe Pharmaceuticals until all data had been entered completely into the database, the data base had been locked, all patients had been allocated to the analysis sets, and the statistical analysis plan had been finalized and signed.

### Measures of efficacy and safety

The primary outcome measure was the change in the total score on the HAM-D 17-item version between day 0 and endpoint. The secondary analysis of the HAM-D scale included an assessment of responder and remission rates. A responder was defined by an HAM-D total score decrease of ≥50% between day 0 and day 42. Remission was defined as an HAM-D total score of ≤7 points at day 42. Other secondary measures of efficacy were the total scores (investigator ratings) on the Clinical Global Impressions Scale (CGI), the Montgomery-Åsberg Depression Rating Scale (MADRS), and the self rating scores on the Beck Depression Inventory (BDI), the 36-item short form health survey (SF-36), and the patients' global self-rating of efficacy.

Safety measures comprised physical examinations, electrocardiograms and laboratory tests before and after the double-blind treatment (blood: erythrocytes, platelets, hemoglobin, hematocrit, leukocytes, neutrophils, eosinophils, basophils, lymphocytes, monocytes, creatinine, glucose, triglycerides, total bilirubin, cholesterol, aspartate transaminase, alanine transaminase, γ-glutamyltransferase, sodium, potassium, calcium, prothrombin time, thromboplastin time and fibrinogen; urinalysis: protein, glucose and blood). Vital signs were tested at each of the three visits. In addition, the patients were thoroughly questioned about adverse events in a general inquiry during each visit. Adverse events were coded using the Medical Dictionary for Regulatory Activities (MedDRA) Version 5.1.

### Statistical analysis

The extent of response to placebo in depression trials tends to be variable and unpredictable [23], and striking differences can arise in the within-group variability of response to treatment. To avoid exposing depressed patients to placebo unnecessarily and to minimize the risk of insufficient statistical power, the study was planned and conducted with an adaptive interim analysis designed to determine whether there was sufficiently convincing evidence, or lack of evidence, of treatment differences to enable early termination or continuation to a second stage with a revised sample size [24]. The interim analysis was performed by a statistican and a programmer who were otherwise not involved in the study.

The primary outcome measure for confirmatory treatment group comparisons of efficacy was the absolute change in HAM-D total score between day 0 and endpoint. The global significance level was α = 0.05 (two-sided). For the interim analysis, the boundaries α_1 _= 0.0299 for rejecting a null hypothesis and α_0 _= 0.30 for termination owing to futility were applied. The global null hypothesis, predicting no difference between any of the treatment group means for the primary outcome measure, was examined first using Bartholomew's test [25,26]. This test is especially suited to the natural ordering of dose groups and is powerful against a variety of outcome patterns [27]. In the event that the global null hypothesis could be rejected, pairwise comparisons between the active treatment groups and placebo were performed using two-sample t-tests. Owing to the unequal sample sizes in the active groups and the placebo group, the Welch-Satterthwaite version of the t-test was used [28]. Application of this testing strategy with local type I error rate α_1 _= 0.0299 in the interim analysis guarantees control of the multiple type I error rate α = 0.05 for the confirmatory test problem for the primary variable [29]. Analysis of the secondary variables was descriptive and hence no adjustments were made for the total number of comparisons. Two-sided p-values are reported throughout.

The relationship between treatment efficacy and the severity of depression before the start of treatment was investigated by explorative subgroup analyses conducted on data from subgroups of patients with initial HAM-D total scores split at the 25^th ^percentile (<20 and ≥20), the median (<23 and ≥23) and the 75^th ^percentile (<25 and ≥25); and on CGI item 1 severity of illness "moderately ill or less severe" and "markedly ill or more severe".

The primary analysis of efficacy was performed on the full analysis set (FAS) of all randomized patients for whom some post-baseline efficacy data was available. For these efficacy analyses, the last observation was carried forward for patients who terminated the trial prematurely. Hence, the reported evaluations and results correspond to baseline-to-endpoint analyses. An additional analysis of the primary outcome measure was performed on the per protocol analysis set (PP), which included all randomized patients with no major protocol deviations. Safety analyses were based on the safety analysis set (SAS), which included all patients who took at least one dose of the study medication after random assignment.

The power was estimated using Monte Carlo simulations (10,000 replications), suited to evaluating the global null hypothesis by Bartholomew's test followed by pairwise t-tests based on a closed test procedure. The standard error for the estimated power was thus approximately 0.3% for a power of 90%. The calculations were based on the assumption of a common standard deviation of 6 points in the HAM-D score, estimated from previous depression studies [2-4].

For sample sizes per group of 120 (WS^® ^5570 600 mg/day), 120 (WS^® ^5570 1200 mg/day) and 80 (placebo), assuming a profile of the difference in expected values from the placebo group of 3, 3 and 0 for WS^® ^5570 600 mg/day, and WS^® ^5570 1200 mg/day and placebo, respectively, and using Bartholomew's test, the power to reject the global null hypothesis was 92.6% and the power for additional rejection of a null hypothesis referring to the comparison between placebo and an active treatment group was 88.5%. Further simulations over a range of assumed dose-response shapes for the primary outcome variable led to similar conclusions.

## Results

### Patient accountability

**Figure 1:**
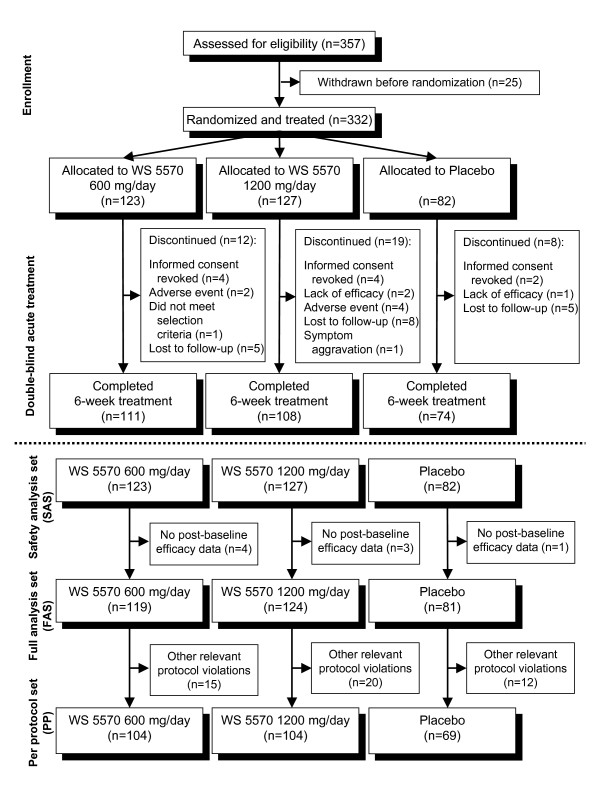
Patient accounting and datasets for analysis.

An overview of the disposition of patients is shown in Figure 1. After giving written informed consent, 357 patients underwent a screening examination to determine their eligibility for the trial, and 332 patients were randomized and treated: 123, 127, and 82 patients received at least one dose of WS^® ^5570 600 mg/day, WS^® ^5570 1200 mg/day or placebo, respectively. These 332 patients constituted the SAS and were analyzed for safety.

Of the 332 patients randomized, 293 completed the 6-week acute treatment: 111 (90.2%) in the WS^® ^5570 600 mg/day group, 108 (85.0%) in the WS^® ^5570 1200 mg/day group and 74 (90.2%) in the placebo group. In total, 12 (9.8%), 19 (15.0%) and 8 (9.8%) patients discontinued prematurely in the WS^® ^5570 600 mg/day, WS^® ^5570 1200 mg/day and placebo treatment groups, respectively. The mean/median times to drop-out for these patients were 24.9 days/25 days, 23.5 days/26 days and 30.8 days/27 days, respectively. The primary reason for early withdrawal was "lost to follow-up" (4.1% for WS^® ^5570 600 mg/day, 6.3% for WS^® ^5570 1200 mg/day and 6.1% for placebo).

Eight patients had no post-baseline efficacy data and were thus excluded from the efficacy analyses: 4 in the WS^® ^5570 600 mg/day group, 3 in the WS^® ^5570 1200 mg/day group and 1 in the placebo group. The remaining 324 patients constituted the FAS and were analyzed for efficacy.

In addition to the 8 patients with no post-baseline efficacy data, there were 15, 20 and 12 patients with relevant protocol violations in the WS^® ^5570 600 mg/day, WS^® ^5570 1200 mg/day and placebo treatment groups, respectively. Decisions as to the relevance of the protocol deviations were made before the code was broken. Premature withdrawal was the most frequent relevant protocol violation in all treatment groups, followed by insufficient treatment compliance. The percentage of non-compliant patients was slightly higher in the WS^® ^5570 1200 mg/day group (18.9%) than in the WS^® ^5570 600 mg/day (15.4%) and the placebo groups (15.9%). The PP analysis set comprised the 277 patients without relevant protocol violations: 104 each in the WS^® ^5570 groups and 69 in the placebo group.

### Baseline characteristics

Demographic and clinical measures at baseline are shown in Table 1. There were no significant differences in demographic characteristics among the three treatment groups. For the HAM-D, MADRS and BDI, a higher value indicates more severe impairment, whereas for the SF-36 a lower score indicates more severe impairment. The HAM-D total scores (primary outcome measure) were comparable among all three groups at baseline. The CGI assessment at baseline did not indicate a higher degree of pathology in the placebo group than in the two WS^® ^5570 groups, whereas for the MADRS and SF-36 the patients in the placebo group exhibited slightly more severe baseline impairment than those in the two WS^® ^5570 groups, though these differences were not significant. For the BDI, the slightly more severe baseline impairment exhibited for the placebo group compared to the two WS^® ^5570 groups was statistically significant.

### Efficacy

Bartholomew's test yielded a p-value of p < 0.001<α_1 _= 0.0299, enabling rejection of the global null hypothesis predicting no difference in mean values between the treatment groups. Thus, the primary objectives of the trial were fulfilled, rendering it unnecessary to perform a second stage.

**Figure 2:**
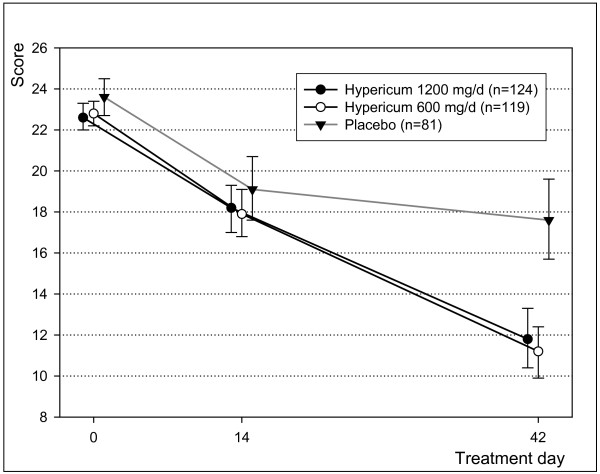
Change in mean total HAM-D score in patients suffering a major depressive episode over the 6-week treatment with WS^® ^5570 or placebo (full analysis set, means and 95% confidence intervals).

WS^® ^5570 600 mg/day and WS^® ^5570 1200 mg/day were demonstrated to be significantly superior to placebo in reducing the HAM-D total score after 42 days of acute treatment (FAS: test statistic = 0.09, p < 0.001; PPS: test statistic = 0.08, p < 0.001). Average total HAM-D scores decreased monotonically in all three groups between the start of randomized treatment and day 42 (Figure 2). Table 2 compares the mean HAM-D scores at baseline and after 14 and 42 days of treatment. The mean HAM-D total score was reduced approximately twice as much for the 600 mg/day and 1200 mg/day WS^® ^5570 groups as for the placebo group. In all three groups, median HAM-D scores decreased by 4 points over the first two weeks. However, patients randomized to either dose of WS^® ^5570 continued to improve between day 14 and day 42 at a rate comparable to that of the initial two weeks, whereas the study participants in the placebo group showed only a small additional average improvement beyond day 14. No significant difference was observed between the WS^® ^5570 600 mg/day and WS^® ^5570 1200 mg/day treatment groups. The median decrease in HAM-D scores between day 42 and day 0 was 13 points for both WS^® ^5570 groups and 3 points for the placebo group. The results obtained from the PP analysis were consistent with those of the FAS analysis.

Subgroup analyses were performed to assess the influence of the pre-treatment severity of depression on the change in HAM-D total score under treatment with WS^® ^5570 or placebo. The subgroups were based on initial HAM-D total scores split at the 25^th ^percentile (<20 and ≥ 20), the median (<23 and ≥ 23) and the 75^th ^percentile (<25 and ≥ 25); and on CGI item 1 severity of illness "moderately ill or less severe" and "markedly ill or more severe". The results are shown in Table 3. Owing to the different and in some cases small sample sizes of the subgroups, the p-values for these analyses are to be interpreted with caution. WS^® ^5570 was consistently more effective than placebo in patients with both less severe and more severe baseline impairment. This applied to all the cutoffs between "lower" and "higher" baseline severity we investigated. The relationship between baseline demographic factors and recurrence of depression on improvement in HAM-D total score between baseline and the end of randomized acute treatment was also investigated. WS^® ^5570 was consistently more effective than placebo in all subgroups investigated; no systematic relationship between treatment efficacy and age, sex, or recurrence of depression was observed.

Secondary efficacy outcome measures are shown in Table 4. Responders were defined as patients with ≥50% decrease in HAM-D total score between day 0 and day 42. The responder rates were approximately 70% and 61% in the WS^® ^5570 600 mg/day and WS^® ^5570 1200 mg/day groups, respectively, whereas the responder rate in the placebo group was approximately 32% (FAS: χ^2 ^= 27.55, df = 1; p < 0.001 for WS^® ^5570 600 mg/day vs placebo; χ^2 ^= 16.70, df = 1; p < 0.001 for WS^® ^5570 1200 mg/day vs placebo). The clinically important criterion of remission was defined as a HAM-D total score ≤7 points at end of treatment. The number of patients who met this criterion was about 33% in the WS^® ^5570 600 mg/day group, about 40% in the WS^® ^5570 1200 mg/day group and about 15% in the placebo group (FAS, χ^2 ^= 8.16, df = 1; p = 0.004 for WS^® ^5570 600 mg/day versus placebo; χ^2 ^= 15.11, df = 1; p < 0.001 for the comparison of WS^® ^5570 1200 mg/day versus placebo; χ^2 ^= 1.49, df = 1; p = 0.22 for the comparison of WS^® ^5570 600 mg/day and WS^® ^5570 1200 mg/day). Results obtained for the PP analysis were analogous.

All items of the CGI showed substantially greater improvements in the two WS^® ^5570 groups than in placebo (Table 4). In about 40% of the patients treated with the herbal extract and 17% of those randomized to placebo, the severity of depression was rated as having been alleviated by at least two categories (e.g. from "markedly ill" to "mildly ill", or from "moderately ill" to "borderline mentally ill"; FAS). More than 60% of patients in both WS^® ^5570 groups and about 31% in the placebo group were much or very much improved. In approximately 35% of the patients treated with WS^® ^5570 and 16% of the placebo group, the investigator observed a "marked therapeutic effect" of the study drug. All pairwise comparisons between WS^® ^5570 600 mg/day or WS^® ^5570 1200 mg/day and placebo showed two-sided p-values of p < 0.01; χ^2 ^= 7.63, df = 1. The differences between the CGI results in the two WS^® ^5570 groups were marginal.

All three treatment groups showed comparable decreases in MADRS mean total score of about 5 points between day 0 and day 14 (Table 4). While the MADRS total scores continued to decrease in the WS^® ^5570 groups between days 14 and 42, patients randomized to placebo showed only marginal changes between the visits at day 14 and day 42. The score reductions were approximately twice as great in the two WS^® ^5570 groups as in the placebo group at treatment end (t = 4.75, df = 159; p < 0.001 for pairwise comparisons of WS^® ^5570 600 mg/day vs placebo and t = 3.69, df = 158; p < 0.001 for WS^® ^5570 1200 mg/day vs placebo). At day 14 as well as at day 42, there were negligible differences between WS^® ^5570 600 mg/day and WS^® ^5570 1200 mg/day in the change of MADRS total score against baseline.

To supplement the observer ratings of severity of depression obtained with the HAM-D and the MADRS, the BDI self-rating scale was administered at baseline and at end of treatment. With average score decreases around 8 points in the two WS^® ^5570 groups versus 3.7 points in the placebo group, patients randomized to WS^® ^5570 subjectively reported substantially greater symptom alleviation than those who received placebo during the double-blind acute treatment. This corresponded to average score decreases of approximately 35% for both WS^® ^5570 groups and 14.2% for placebo. The pairwise comparisons between WS^® ^5570 600 mg/day and placebo or WS^® ^5570 1200 mg/day and placebo showed p-values of p < 0.001 with t = 3.90, df = 179 and t = 3.62, df = 185. There were no significant differences between the average BDI self-ratings of the two WS^® ^5570 groups.

The 36 items of the SF-36 can be divided into the two summary measures physical health and mental health. Following comparable baseline values for the mental health summary measure among the three treatment groups (Table 1), the patients randomized to WS^® ^5570 showed average improvements over baseline for the mental health summary measure about twice as great as those in the placebo group (t = 4.74, df = 190; p < 0.001 for WS^® ^5570 600 mg/day vs placebo; t = 3.77, df = 201; p < 0.001 for WS^® ^5570 1200 mg/day vs placebo).

At the end of the 6-week treatment, a global self-rating of efficacy was obtained from 107 patients in the WS^® ^5570 600 mg/day group, 105 patients in the WS^® ^5570 1200 mg/day group and 69 patients in the placebo group. The efficacy of the investigational treatment was rated "good" or "very good" for 76 (71.0%) randomized to WS^® ^5570 600 mg/day, 67 (63.8%) treated with WS^® ^5570 1200 mg/day and 23 (33.3%) in the placebo group (chi-square test: WS^® ^5570 600 mg/day vs placebo p < 0.001; WS^® ^5570 1200 mg/day vs placebo p < 0.001). Treatment efficacy was rated "bad" or "very bad" by 18 (16.8%) patients in the WS^® ^5570 600 mg/day group, 25 (23.8%) in the WS^® ^5570 1200 mg/day group, and 36 (52.2%) randomized to placebo.

### Safety and tolerability

The adverse events that occurred during the treatment phase are shown in Table 5. The reported p-values were calculated using Fisher's exact test. The most frequently reported adverse events were related to gastrointestinal disorders, affecting 19.5% (24 of 123) of the patients in the WS^® ^5570 600 mg/day group, 23.6% (30 of 127) of those in the WS^® ^5570 1200 mg/day group and 15.9% (13 of 82) of those in the placebo group. None of the frequencies showed any dose-related relationship. Serious adverse events were reported in 3 patients after randomization: 1 patient in the WS^® ^5570 600 mg/day group (with an event of tendon rupture attributable to accidental injury) and 2 in the WS^® ^5570 1200 mg/day group (with events of depression aggravation and acute stress disorder, attributable to the underlying disease and not tolerability issues). One serious adverse event (suicide attempt) occurred after screening but before randomization. All serious adverse events were classified as serious owing to hospitalization. There were few premature withdrawals due to adverse events: 2 (1.6%) patients in the WS^® ^5570 600 mg/day group (with events of erythema and herpetic stomatitis) and 4 (3.2%) in the WS^® ^5570 1200 mg/day group (with events of upper abdominal pain, stomatitis, acute stress disorder and metorrhagia).

Adverse events were assessed for causal relationship to the treatment under investigation before unblinding. Such events were considered potentially related to the treatment for 30 (24.4%), 31 (24.4%) and 13 (15.9%) patients in the WS^® ^5570 600 mg/day, 1200 mg/day and placebo groups, respectively. Among these potentially attributable adverse events, the most frequent were gastrointestinal disorders, which were assessed as possibly related in all three treatment-groups. No dose-related relationship was observed. All other adverse events for which a causal relationship with the investigational treatments could not be excluded occurred in not more than two patients in any group. Two women treated with WS^® ^5570 experienced metorrhagia and one reported menorrhagia; these adverse events have previously been associated with the administration of *Hypericum *extract. Furthermore, one case of mildly increased sensitivity to sunlight and another case of moderate sunburn were assessed as related to WS^® ^5570 treatment; photosensitivity reactions are among the known side effects of *Hypericum *extract.

None of the laboratory safety parameters showed any relevant changes in the mean, and incidences of clinically relevant deviations from the reference ranges were comparable between the study groups. No noteworthy changes were observed in vital signs, physical examination or electrocardiograms.

## Discussion

### Efficacy

The results of this study demonstrate the superior antidepressant efficacy of WS^® ^5570 600 mg/day (in one dose) and of WS^® ^5570 1200 mg/day (in two daily doses) compared to placebo in the treatment of patients with a mild or moderate major depressive episode after 6 weeks of treatment. Over the first two weeks, the mean decreases in HAM-D scores were nearly the same, about 4 points in all three groups. Over the full 6 weeks, mean decreases in HAM-D scores were approximately 11 points for both WS^® ^5570 groups and 6 points for the placebo group; the difference of 5 points was significant (FAS: t = 5.26, df = 145; p < 0.001 for WS^® ^5570 600 mg/day vs placebo; t = 4.32, df = 159; p < 0.001 for WS^® ^5570 1200 mg/day vs placebo). The findings of the analyses with the FAS were fully supported by the PP analyses.

On a descriptive level, the antidepressant effects of WS^® ^5570 and placebo were comparable during the first two weeks of treatment, indicating that without a placebo run-in phase, factors other than the pharmacological action of *Hypericum *extract may have initially contributed to the observed relief of depressive symptoms. However, the majority of patients randomized to placebo showed only limited or no improvement between day 14 and day 42, whereas patients treated with WS^® ^5570 600 mg/day and WS^® ^5570 1200 mg/day continued to improve at an almost constant rate until the end of this study period, so the treatment group differences observed at the end of the 6-week acute phase can be attributed to the pharmacological effect of the herbal extract.

The effect size observed in this study was greater than that observed in a study with 300 mg WS^® ^5570 three times daily (i.e. WS^® ^5570 900 mg/day) and placebo [4]. Although the mean HAM-D scores at day 42 for the WS^® ^5570 900 mg/day group in that study were similar to the scores observed for WS^® ^5570 600 mg/day and WS^® ^5570 1200 mg/day in the present study, the mean scores for patients randomized to placebo were lower than those of the placebo patients observed in the present study.

The difference of about 30% in responder rates (69.8% in the WS^® ^5570 600 mg/day group, 61.3% in the WS^® ^5570 1200 mg/day group and 31.1% in the placebo group) was significant (FAS: χ^2 ^= 27.55, df = 1; p < 0.001 for WS^® ^5570 600 mg/day vs placebo; χ^2 ^= 16.70, df = 1; p < 0.001 for WS^® ^5570 1200 mg/day vs placebo). The differences in remission rates (32.8% in the WS^® ^5570 600 mg/day group, 40.3% in the WS^® ^5570 1200 mg/day group and 14.8% in the placebo group) were also significant (FAS, χ^2 ^= 8.16, df = 1; p < 0.01 for the comparison of the WS^® ^5570 600 mg/day group versus placebo and χ^2 ^= 15.11, df = 1; p < 0.001 for the comparison of the WS^® ^5570 1200 mg/day group versus placebo). These effects were also greater than those observed between 300 mg WS^® ^5570 three times daily and placebo [4].

Other secondary measures of treatment efficacy, i.e. the CGI and MADRS observer rating scales, the BDI self-rating scale, the SF-36 and the patient global self-rating, showed results consistent with those obtained for the primary outcome measure: statistically significant changes between baseline and day 42 for WS^® ^5570 600 mg/day and 1200 mg/day compared to placebo.

Descriptively, the remission rates were about 8% greater for the WS^® ^5570 1200 mg/day group than for the WS^® ^5570 600 mg/day group, i.e. there is a possible dose-dependency. For most of the outcome measures assessed, only minor differences in efficacy were observed between WS^® ^5570 600 mg/day and 1200 mg/day. This applied to the analysis data sets as a whole as well as to subgroups defined by baseline severity of depression. Thus, the dose of WS^®^5570 600 mg/day can be considered sufficiently efficacious; doubling the dosage increases the chances of therapeutic success only slightly.

### Safety and tolerability

For all three treatment-groups, the highest frequencies of adverse events were observed during the first two weeks of acute treatment. Because this effect was observed in all three treatment groups and in the absence of a placebo run-in phase, it is likely to have been related to a nonspecific, non-drug-related effect based on the patients' and investigators' expectations or to the patients' initial severity of depression (known to increase vulnerability to various kinds of adverse experiences).

During the 6-week acute treatment, the WS^® ^5570 600 mg/day and 1200 mg/day groups showed a slightly higher frequency of adverse events than the placebo group (0.012 and 0.013 versus 0.008 events per day of exposure, respectively). However, there were no meaningful differences in incidence rates in any of the System Organ Classes of the MedDRA system. Furthermore, when only potentially attributable events were considered, marginal treatment group differences in adverse event incidence were observed: 0.007, 0.007 and 0.005 events per day of exposure during acute treatment for WS^® ^5570 600 mg/day, 1200 mg/day and placebo groups, respectively. The WS^®^5570 1200 mg/day dose was as well tolerated as the 600 mg/day dose.

Potentially attributable adverse events included gastrointestinal disorders, menstrual complications and photosensitivity reactions. The events occurred with low frequencies, and all three types of events have been associated with *Hypericum *extract as rare, transient adverse reactions. Aside from these three types, the results of this study do not point to any drug-specific adverse events. A systematic literature review of the adverse effects of St. John's wort is given by Hammerness et al. [30].

WS^® ^5570 had no meaningful influence on laboratory measures, physical findings, vital signs or ECG parameters.

## Conclusion

This trial demonstrates that *Hypericum *extract WS^® ^5570 600 mg/day (given once daily) and WS^® ^5570 1200 mg/day (divided into two daily doses) are both efficacious in the treatment of mild or moderate unipolar major depression. This principal finding was consistent across several validated investigator- and self-rating scales, across the participating centers, and for different analysis data sets (including or excluding patients with major protocol violations). Decreases in average HAM-D total score versus baseline were approximately 6 and 5 points greater after the 6-week treatment for WS^® ^5570 600 mg/day and WS^® ^5570 1200 mg/day, respectively, than for placebo. The responder rates of 70% and 61% versus 32% and the remission rates of 33% and 40% versus 15% for WS^® ^5570 600 mg/day, WS^® ^5570 1200 mg/day and placebo, respectively, also underscore the clinical relevance of the observed effect. The interim analysis showed statistical significance versus placebo in favor of both treatment regimens of WS^®^5570, allowing rejection of the null hypotheses in accordance with the biometrical design pre-specified in the protocol. Hence, the study could be terminated early owing to the proof of efficacy for both active treatment groups.

The primary outcome measure, the HAM-D total score change versus baseline after 6 weeks, and the psychiatric scales administered as secondary efficacy measures, indicate no meaningful differences in efficacy between WS^® ^5570 600 mg/day (in one dose) and 1200 mg/day (in two doses). However, more patients in the 1200 mg/day dose met the clinically important criterion of remission (HAM-D total score ≤7 points at treatment end) in descriptive analyses.

WS^® ^5570 at doses of 600 mg/day and 1200 mg/day was well tolerated. Treatment with *Hypericum *extract was associated with a slightly higher rate of adverse events than treatment with placebo, but these differences were minor, particularly for potentially attributable events. All potentially attributable adverse events were non-serious and transient and reflected the known profile of adverse events for *Hypericum *extract. Thus, the trial did not reveal any previously unknown risks in treatment with *Hypericum *extract.

Although the WS^® ^5570 1200 mg/day dose did not prove to be significantly more effective than the 600 mg/day dose, the doubled dose was equally safe and well tolerated.

In conclusion, *Hypericum *extract WS^® ^5570 600 mg/day and 1200 mg/day were shown to be safe and more efficacious than placebo in the 6-week treatment of patients suffering from a mildly to moderately intense major depressive episode.

## Competing interests

AD and MK are employees of Dr. Willmar Schwabe Pharmaceuticals.

SK, IA and AS received consultancy fees from Dr. Willmar Schwabe Pharmaceuticals

## Authors' contributions

SK participated in study design and interpretation of data and drafted the manuscript.

IA participated in study coordination and interpretation and drafted the manuscript

AS participated in study design and coordination and drafted the manuscript

AD participated in study design, coordination and interpretation and drafted the manuscript.

MK participated in study design and interpretation, performed the analysis and drafted the manuscript.

All authors read and approved the final manuscript.

## Pre-publication history

The pre-publication history for this paper can be accessed here:



## Figures and Tables

**Table 1:** Demographic and clinical characteristics at baseline for patients with a major depressive episode (full analysis set)

**Characteristic**		**WS^® ^5570 600 mg/day N = 119**	**WS^® ^5570 1200 mg/day N = 124**	**Placebo N = 81**	**p-value**
Sex:					0.13*
Female	n (%)	67 (56.3)	82 (66.1)	56 (69.1)	
Male	n (%)	52 (43.7)	42 (33.9)	25 (30.9)	
					
Age (years)	Mean ± SD	46.3 ± 11.5	46.1 ± 10.7	46.9 ± 11.8	0.69^§^
					
HAM-D total score	Mean ± SD	22.8 ± 3.3	22.6 ± 3.8	23.6 ± 4.2	0.31
					
CGI severity of illness:					0.20*
Mildly or moderately ill	n (%)	67 (56.3)	62 (50.0)	46 (56.8)	
Markedly or severely ill	n (%)	52 (43.7)	62 (50.0)	35 (43.2)	
					
MADRS	Mean ± SD	27.2 ± 5.5	26.3 ± 6.0	28.0 ± 6.2	0.09^§^
					
BDI	Mean ± SD	23.2 ± 7.9	23.3 ± 8.0	25.7 ± 8.9	0.05^§^
					
SF-36 summary measure mental health	Mean ± SD	25.1 ± 11.7	27.7 ± 13.3	23.7 ± 11.1	0.16^§^

For the HAM-D, MADRS and BDI, a higher value indicates more severe impairment whereas for the SF-36, a lower score indicates more severe impairment.

*Two-sided chi-square test

^§^Two-sided Kruskal-Wallis test

**Table 2:** Comparison of HAM-D scores for patients with a major depressive episode over 6-week treatment with WS^® ^5570 or placebo (full analysis set; last observation carried forward)

**HAM-D measure**		**WS^® ^5570 600 mg/day N = 119**	**WS^® ^5570 1200 mg/day N = 124**	**Placebo N = 81**	**p-value**	
					
					**WS^® ^5570 600 mg/day vs placebo**	**WS^® ^5570 1200 mg/day vs placebo**
Total score (mean ± SD)	Day 0	22.8 ± 3.3	22.6 ± 3.8	23.6 ± 4.2	0.14	0.09
	Day 14	17.9 ± 6.3	18.2 ± 6.6	19.1 ± 20	0.23	0.34
	Day 42	11.2 ± 7.0	11.8 ± 8.3	17.6 ± 8.8	<0.001	<0.001
Difference vs day 0 (mean ± SD)	Day 14	-4.9 ± 5.1	-4.5 ± 4.7	-4.5 ± 5.4	0.65	0.95
	Day 42	-11.6 ± 6.4	-10.8 ± 7.3	-6.0 ± 8.1	<0.001	<0.001
Mean reduction vs day 0 (%)	Day 14	22.0%	20.9%	16.7%	0.54	0.77
	Day 42	57.9%	57.1%	16.7%	<0.001	<0.001

**Comparison vs placebo for difference day 42 vs day 0**		**WS^® ^5570 600 mg/day vs placebo**		**WS^® ^5570 1200 mg/day vs placebo**		
Differences in means*		-5.6		-4.8		
95% confidence interval		[-7.7, -3.6]		[-6.9, -2.6]		
p-value		<0.001		<0.001		

*A negative difference denotes a more favorable outcome in the treatment group named first.

**Table 3:** Relative decreases in HAM-D scores for subgroups of patients with a major depressive episode after 6 weeks of treatment with WS^® ^5570 or placebo (full analysis set, last observation carried forward)

**Subgroup based on baseline score**		**Mean % decrease ± SD, number of patients**			**p-value**	
		
		**WS^® ^5570 600 mg/day N = 119**	**WS^® ^5570 1200 mg/day N = 124**	**Placebo N = 81**	**WS^® ^5570 600 mg/day vs placebo**	**WS^® ^5570 1200 mg/day vs placebo**
Baseline HAM-D total score cutoff 20 (split at 25^th ^percentile)	<20	54.2 ± 26.3, 25	56.0 ± 29.9, 31	32.7 ± 40.5, 14	0.09	0.07
	≥ 20	51.1 ± 28.3, 94	46.4 ± 32.5, 93	24.4 ± 33.6, 67	<0.001	<0.001
Baseline HAM-D total score cutoff 23 (split at median)	<23	55.5 ± 28.1, 56	53.1 ± 29.7, 64	26.8 ± 38.8, 37	<0.001	<0.001
	≥ 23	48.4 ± 27.4, 63	44.2 ± 33.9, 60	25.1 ± 31.4, 44	<0.001	<0.01
Baseline HAM-D total score cutoff 25 (split at 75^th ^percentile)	<25	53.6 ± 27.3, 83	52.8 ± 30.2, 85	28.6 ± 37.7, 49	<0.001	<0.001
	≥ 25	47.4 ± 28.9, 36	40.2 ± 34.5, 39	21.7 ± 29.8, 32	<0.001	0.02
Baseline CGI item 1, severity of illness	<5*	51.2 ± 28.7, 67	49.9 ± 31.5, 62	28.0 ± 36.1, 46	<0.001	<0.01
	≥ 5^§^	52.4 ± 26.9, 52	47.7 ± 32.7, 62	23.1 ± 33.3, 35	<0.001	<0.001

* "Moderately ill or less severe"

^§ ^"Markedly ill or more severe"

**Table 4:** Secondary efficacy measures for patients with a major depressive episode treated over 6-weeks with WS^® ^5570 or placebo (full analysis set, last observation carried forward)

				**p-value**	
				
Secondary efficacy measure*	**WS^® ^5570 600 mg/day N = 119**	**WS^® ^5570 1200 mg/day N = 124**	**Placebo N = 81**	**WS^® ^5570 600 mg/day vs placebo**	**WS^® ^5570 1200 mg/day vs placebo**
			**n (%)**		

Responder rate (decrease ≥ 50% in total HAM-D score between day 0 and day 42)	83 (69.8)	76 (61.3)	26 (31.1)	<0.001	<0.001
Remission rate (≤ 7 points in total HAM-D score at day 42)	39 (32.8)	50 (40.3)	12 (14.8)	<0.01	<0.001
CGI, item 1:					
improved by ≥ 2 categories	46 (38.7)	54 (43.6)	14 (17.3)	0.001	<0.001
CGI, item 2:					
much or very much improved	76 (63.9)	77 (62.1)	25 (30.9)	<0.001	<0.001
CGI, item 3:					
marked therapeutic effect	40 (33.6)	47 (37.9)	13 (16.1)	0.006	<0.001

			**Mean ± SD**		

MADRS absolute change					
day 14 – day 0	-5.3 ± 7.7	-4.4 ± 5.5	-5.0 ± 6.6	0.80	0.46
day 42 – day 0	-13.3 ± 9.3	-11.8 ± 9.3	-6.5 ± 10.4	<0.001	<0.001
MADRS relative decrease (%)					
day 14 – day 0	19.5 ± 26.7	17.5 ± 22.8	18.1 ± 25.1	0.72	0.85
day 42 – day 0	48.9 ± 31.6	45.6 ± 34.2	22.2 ± 36.6	<0.001	<0.001
BDI absolute change					
(day 42 – day 0)	-8.3 ± 8.5	-8.0 ± 9.1	-3.7 ± 7.9	<0.001	<0.001
BDI relative change (%)					
day 42 – day 0	34.6 ± 34.0	34.1 ± 36.8	14.2 ± 36.9	<0.001	<0.001
SF-36 summary measures (day 42 – day 0)					
Physical health	3.8 ± 18.3	5.8 ± 16.3	2.2 ± 13.7	0.47	0.09
Mental health	18.1 ± 18.7	16.5 ± 21.9	6.8 ± 14.6	<0.001	<0.001

*For the HAM-D, the MADRS and the BDI, higher values indicate more severe impairment whereas for the SF-36, lower values indicate more severe impairment

**Table 5:** Adverse events reported during the 6-week treatment with WS^® ^5570 or placebo (safety analysis set)

**Type of adverse event (MedDRA System Organ Class)**	Number (%) of patients with adverse events*			**p-value**	
	
	**WS^® ^5570 600 mg/day N = 123**	**WS^® ^5570 1200 mg/day N = 127**	**Placebo N = 82**	**WS^® ^5570 600 mg/day vs placebo**	**WS^® ^5570 1200 mg/day vs placebo**
**All adverse events**	**49 (39.8)**	**50 (39.4)**	**22 (26.8)**	**0.07**	**0.07**
Ear and labyrinth disorders	3 (2.4)	2 (1.6)	1 (1.2)	0.65	1.00
Eye disorders	0 (0.0)	1 (0.8)	0 (0.0)	1.00	1.00
Gastrointestinal disorders	24 (19.5)	30 (23.6)	13 (15.9)	0.58	0.22
General disorders and administration site conditions	2 (1.6)	2 (1.6)	0 (0.0)	0.52	0.52
Infections and infestations	7 (5.7)	4 (3.2)	2 (2.4)	0.32	1.00
Injury, poisoning and procedural complications	1 (0.8)	1 (0.8)	1 (1.2)	1.00	1.00
Investigations	1 (0.8)	0 (0.0)	0 (0.0)	1.00	1.00
Metabolism and nutrition disorders	1 (0.8)	1 (0.8)	1 (1.2)	1.00	1.00
Musculosceletal and connective tissue disorder	1 (0.8)	2 (1.6)	1 (1.2)	1.00	1.00
Nervous system disorder	6 (4.9)	6 (4.7)	2 (2.4)	0.48	0.49
Psychiatric disorders	2 (1.6)	2 (1.6)	0 (0.0)	0.52	0.52
Renal and unrinary disorders	1 (0.8)	0 (0.0)	0 (0.0)	1.00	1.00
Reproductive system and breast disorders	1 (0.8)	2 (1.6)	0 (0.0)	1.00	0.52
Respiratory, thoracic and mediastinal disorders	4 (3.3)	5 (3.9)	2 (2.4)	1.00	0.71
Skin and subcutaneous disorders	4 (3.3)	2 (1.6)	4 (4.9)	0.72	0.21
Vascular disorders	1 (0.8)	1 (0.8)	0 (0.0)	1.00	1.00

Adverse events were coded using the Medical Dictionary for Regulatory Activities (MedDRA) Version 5.1.

*Patients may have experienced more than one adverse event.

## References

[B1] Laakmann G, Dienel A, Kieser M (1998). Clinical significance of hyperforin for the efficacy of *Hypericum *extracts on depressive disorders of different severities. Phytomedicine.

[B2] Laakmann G, Schüle C, Baghai T, Kieser M (1998). St. John's wort in mild to moderate depression: the relevance of hyperforin for the clinical efficacy. Pharmacopsychiatry.

[B3] Kalb R, Trautmann-Sponsel RD, Kieser M (2001). Efficacy and tolerability of *Hypericum *extract WS5572 versus placebo in mildly to moderately depressed patients. Pharmacopsychiatry.

[B4] Lecrubier Y, Clerc G, Didi R, Kieser M (2002). Efficacy of St. John's wort extract WS5570 in major depression: a double-blind, placebo-controlled trial. Am J Psychiatry.

[B5] Vorbach EU, Arnoldt KH, Hübner WD (1997). Efficacy and tolerability of St. John's Wort Extract LI 160 versus imipramine in patients with severe depressive episodes according to ICD-10. Pharmacopsychiatry.

[B6] Schrader E, Meier B, Brattström A (1998). Hypericum treatment of mild-moderate depression in a placebo-controlled study. A prospective, double-blind, randomized, placebo-controlled, multicentre study. Hum Psychopharmacol.

[B7] Kasper S, Trautmann-Sponsel RD (2001). St John's wort extract: an effective and well-tolerated antidepressant. Primary Care Psychiatry.

[B8] Linde K, Knüppel L (2005). Large-scale observational studies of hypericum extracts in patients with depressive disorders-a systematic review. Phytomedicine.

[B9] Hübner WD, Lande S, Pozuweit H (1994). Hypericum treatment of mild depressions with somatic symptoms. J Geriatr Psychiatry Neurol.

[B10] Sommer H, Harrer G (1994). Placebo-controlled double-blind study examining the effectiveness of an Hypericum preparation in 105 mildly depressed patients. J Geriatr Psychiatry Neurol.

[B11] Shelton RC, Keller MB, Gelenberg A, Dunner DL, Hirschfeld R, Thase ME, Russell J, Lydiard RB, Crits-Cristoph P, Gallop R, Todd L, Hellerstein D, Goodnick P, Keitner G, Stahl SM, Halbreich U (2001). Effectiveness of St John's Wort in Major Depression. JAMA.

[B12] Hypericum Depression Trial Study Group (2002). Effect of Hypericum perforatum (St John's Wort) in Major Depressive Disorder. JAMA.

[B13] Fava M, Alpert J, Nierenberger AA, Mischoulon D, Otto MW, Zajecka J, Murck H, Rosenbaum JF (2005). A Double-blind, Randomized Trial of St John's Wort, Fluoxetine, and Placebo in Major Depressive Disorder. J Clin Psychopharmacol.

[B14] Linde K, Berner M, Egger M, Mulrow C (2005). St John's wort for depression: Meta-analysis of randomised controlled trials. Br J Psychiatry.

[B15] Kasper S, Dienel A (2002). Cluster analysis of symptoms during antidepressant treatment with Hypericum extract in mildly to moderately depressed out-patients. A meta-analysis of data from three randomized, placebo-controlled trials. Psychopharmacology.

[B16] Linde K, Mulrow CD, Berner M, Egger M (2005). St John's wort for depression. Cochrane Database Syst Rev.

[B17] Müller WE, Rolli M, Schäfer C, Hafner U (1997). Effects of Hypericum extract (LI 160) in biochemical models of antidepressant activity. Pharmacopsychiatry.

[B18] Perovic S, Müller WE (1995). Pharmacological profile of Hypericum extract. Effect on serotonin uptake by postsynaptic receptors. Arzneimittelforschung.

[B19] Szegedi A, Kohnen R, Dienel A, Kieser M (2005). Acute treatment of moderate to severe depression with hypericum extract WS5570 (St John's wort): randomised controlled double blind non-inferiority trial versus paroxetine. Br Med J.

[B20] German Federal Health Office (BGA) Monograph Hyperici herba (St. John's Wort) Commission E monograph. Bundesanzeiger.

[B21] Rychlik R, Siedentop H, von den Driesch V, Kasper S (2001). St. John's wort extract WS5572 in minor to moderately severe depression. Effectiveness and tolerance of 600 and 1200 mg ingredient daily Fortschr Med Orig.

[B22] American Psychiatric Association (1994). Diagnostic and Statistical Manual of Mental Disorders.

[B23] Linde K, Ramirez G, Mulrow CD, Pauls A, Weidenhammer W, Melchart D (1996). St John's wort for depression-an overview and meta-analysis of randomized clinical trials. Br Med J.

[B24] Bauer P, Koehne K (1994). Evaluation of experiments with adaptive interim analyses. Biometrics.

[B25] Bartholomew DJ (1959). A test of homogeneity for ordered alternatives. Biometrika.

[B26] Bartholomew DJ (1959). A test of homogeneity of means under restricted alternatives. Biometrika.

[B27] Phillips A (1998). A review of the performance of tests used to establish whether there is a drug effect in dose-response studies. Drug Inf J.

[B28] Zimmerman DW (2004). Conditional probabilities of rejecting H_0 _by pooled and separate-variances t tests given heterogeneity of sample variances. Communications in Statistics: Simulation and Computation.

[B29] Kieser M, Bauer P, Lehmacher W (1999). Inference on multiple endpoints in clinical trials with adaptive interim analyses. Biom J.

[B30] Hammerness P, Basch E, Ulbricht C, Barette EP, Foppa I, Basch S, Bent S, Boon H, Ernst E (2003). St. John's Wort: A Systematic Review of Adverse Effects and Drug Interactions for the Consultation Psychiatrist. Psychosomatics.

